# Case report: Unusual episodic myopathy in a patient with novel homozygous deletion of first coding exon of *MICU1* gene

**DOI:** 10.3389/fneur.2022.1008937

**Published:** 2022-11-08

**Authors:** Margarita Sharova, Mikhail Skoblov, Elena Dadali, Nina Demina, Olga Shchagina, Fedor Konovalov, Maria Ampleeva, Inna Sharkova, Sergey Kutsev

**Affiliations:** ^1^Research Centre for Medical Genetics, Moscow, Russia; ^2^Independent Clinical Bioinformatics Laboratory, Moscow, Russia

**Keywords:** MICU1, myopathy with extrapyramidal signs, RNA analysis, molecular mechanism of inherited disease, mitochondrial Ca^2+^ complex

## Abstract

We present a patient with unusual episodes of muscular weakness due to homozygous deletion of exon 2 in the *MICU1* gene. Forty-three patients from 33 families were previously described with homozygous and compound heterozygous, predominantly loss of function (LoF) variants in the *MICU1* gene that lead to autosomal recessive myopathy with extrapyramidal signs. Most described patients developed muscle weakness and elevated CK levels, and half of the patients had progressive extrapyramidal signs and learning disabilities. Our patient had a few severe acute episodes of muscle weakness with minimal myopathy features between episodes and a strongly elevated Creatinine Kinase (CK). Whole exome sequencing (WES) was performed and the homozygous deletion of exon 2 was suspected. To validate the diagnosis, we performed an RNA analysis of all family members. To investigate the possible impact of this deletion on the phenotype, we predicted a new Kozak sequence in exon 4 that could lead to the formation of a truncated MICU1 protein that could partly interact with MCU protein in a mitochondrial Ca^2+^ complex. We suspect that this unusual phenotype of the proband with MICU1-related myopathy could be explained by the presence of the truncated but partly functional protein. This work helps to define the clinical polymorphism of *MICU1* deficiency better.

## Introduction

Myopathy with extrapyramidal signs (OMIM #615673) is a rare autosomal recessive disease due to a pathogenic variant in the *MICU1* gene with a frequency lower than 1/1000000.

The MICU1 protein is a key regulator of the mitochondrial Ca^2+^ uptake complex. MICU1 protein interacts with mitochondrial Ca^2+^ uniporter MCU, which transports elevated cytosolic Ca^2+^ into the mitochondrial matrix and prevents excess mitochondrial Ca^2+^ uptake (1–4). Also, it was shown that differences in mitochondrial membrane potential or altered endoplasmic reticulum Ca^2+^ content could be the cause of increased velocity of Ca^2+^ uptake. Chronic activation of the MCU channel and subsequent elevation of Ca^2+^ in the mitochondrial matrix might lead to moderate mitochondrial stress, as reflected by the fragmented mitochondria phenotype (5).

To date, there are 13 pathogenic variants in the *MICU1* gene described in the HGMD (Human Gene Mutation Database). A clinical description is available for 34 patients with 8 different variants in the *MICU1* gene based on the HGMD database (Table 1). For the other 9 patients, there is only information about the variant type and limited clinical information available. Most patients demonstrated motor and speech delay, learning disabilities, elevated CK and liver transaminases, muscular hypotonia, muscular weakness, and progressive extrapyramidal signs (5–10). The age of onset of muscular weakness ranged from 11 months (5) to 44 years (8). The age of onset of extrapyramidal symptoms such as chorea, tremor, and dystonia ranged from 2 (5) to 40 years (6). Patients with a classical clinical picture of disease were described with loss of function nonsense or canonical splicing site variants in homozygous or compound heterozygous states. Wherein, there is only one family which had an unusual presentation of the disease. The authors described acute attacks of muscular aches, fatigue, and lethargy after physical activity in two cousins with homozygous deletion of the 5′UTR region and the first exon of *MICU1* (10).

**Table 1:** Clinical characteristics of previously described patients.

	**Age of presentation (years)**	**Variant**	**Zygodity**	**Short stature**	**Motor delay**	**Speech delay**	**Learning disabilities**	**Elevated liver transaminases**	**Elevated CK max level**	**Abnormal gait**	**Muscular hypotonia**	**Muscule weakness**	**Extrapyrimidal signs**	**Muscular cramps**	**Calf muscle hypertrophy**	**Ataxia**	**Seizures**	**Microcephaly**	**MRI findings**	**Acute episodes**
Musa F1	9	p.Q185X	homo	yes	yes	yes	yes	yes	yes	no	yes	yes	no	yes	no	n/a	n/a	n/a	n/a	no
Musa F2	2	p.Q185X	homo	yes	yes	yes	no	yes	yes	yes	yes	no	yes	no	no	n/a	no	n/a	yes	no
Musa F3	10	p.Q185X	homo	yes	yes	yes	yes	yes	yes	no	no	yes	no	no	no	n/a	n/a	n/a	n/a	no
Musa F4	11	p.Q185X	homo	no	yes	yes	yes	n/a	yes	no	no	no	no	no	no	n/a	n/a	n/a	n/a	no
Musa F5.1	23	p.Q185X	homo	n/a	yes	yes	yes	yes	yes	no	no	no	yes	yes	yes	n/a	yes	n/a	no	no
Musa F5.2	21	p.Q185X	homo	n/a	yes	yes	yes	yes	yes	no	no	no	yes	yes	yes	n/a	yes	n/a	no	no
Musa F5.3	21	p.Q185X	homo	n/a	yes	yes	yes	yes	yes	no	no	no	yes	yes	yes	n/a	yes	n/a	no	no
Musa F6	2	p.Q185X	homo	yes	yes	yes	n/a	yes	yes	n/a	no	no	no	no	no	n/a	n/a	n/a	n/a	no
Musa F7	4	p.Q185X; Ex9-10del	compound hetero	no	yes	n/a	n/a	yes	yes	yes	yes	yes	no	no	no	n/a	n/a	n/a	n/a	no
Musa F8.1	6	p.Q185X	homo	no	yes	n/a	yes	yes	yes	yes	yes	yes	no	no	no	n/a	n/a	n/a	n/a	no
Musa F8.1	14	p.Q185X	homo	no	yes	n/a	yes	yes	yes	yes	yes	yes	no	no	no	n/a	n/a	n/a	n/a	no
Musa F9	10	p.Q185X	homo	no	yes	yes	yes	yes	yes	no	no	no	no	no	no	n/a	yes	n/a	n/a	no
Musa F10	3	p.Q185X	homo	yes	yes	yes	yes	yes	yes 5175	n/a	yes	yes	yes	no	no	n/a	n/a	n/a	n/a	no
Mojbafan F1	10	c.1295del	homo	n/a	no	yes	n/a	yes	yes	n/a	n/a	n/a	yes	n/a	yes	n/a	n/a	n/a	n/a	no
Bitarafan	44	p.R129X	homo	no	no	no	yes	yes	yes	yes	n/a	yes	yes	n/a	n/a	yes	no	no	no	no
Logan F1	1y 6m	c.1078-1G>C	homo	no	yes	no	yes	n/a	yes 400	n/a	n/a	no	yes	n/a	n/a	no	n/a	yes	no	no
Logan F2.1	1y 10m	c.1078-1G>C	homo	no	yes	no	yes	n/a	yes 2500	n/a	n/a	no	yes	n/a	n/a	no	n/a	no	n/a	no
Logan F2.2	1y 6m	c.1078-1G>C	homo	no	yes	no	no	n/a	yes 2800	n/a	n/a	no	no	n/a	n/a	no	n/a	no	n/a	no
Logan F3.1	1y 6m	c.1078-1G>C	homo	no	yes	yes	yes	n/a	yes 1300	n/a	n/a	yes	yes	n/a	n/a	no	n/a	yes	n/a	no
Logan F3.2	2	c.1078-1G>C	homo	no	yes	no	yes	n/a	n/a	n/a	n/a	yes	yes	n/a	n/a	no	n/a	no	n/a	no
Logan F4	5	c.1078-1G>C	homo	yes	yes	yes	yes	n/a	yes 8000	n/a	n/a	yes	yes	n/a	n/a	no	n/a	no	yes	no
Logan F5	3	c.1078-1G>C	homo	no	yes	yes	yes	n/a	yes 12500	n/a	n/a	no	yes	n/a	n/a	no	n/a	no	no	no
Logan F6	3	c.1078-1G>C	homo	no	yes	yes	yes	n/a	yes 4800	n/a	n/a	yes	yes	n/a	n/a	no	n/a	yes	no	no
Logan F7	2	c.1078-1G>C	homo	yes	yes	no	yes	n/a	yes 4200	n/a	n/a	no	yes	n/a	n/a	no	n/a	yes	n/a	no
Logan F8.1	3	c.1078-1G>C	homo	yes	yes	yes	yes	n/a	yes 8000	n/a	n/a	no	yes	n/a	n/a	yes	n/a	no	no	no
Logan F8.2	2	c.1078-1G>C	homo	no	yes	yes	yes	n/a	yes 1600	n/a	n/a	no	yes	n/a	n/a	no	n/a	yes	yes	no
Logan F9.1	2y 4m	c.741 + 1G>A	homo	yes	no	no	yes	n/a	yes 9000	n/a	n/a	yes	yes	n/a	n/a	yes	n/a	no	yes	no
Logan F9.2	11m	c.741 + 1G>A	homo	no	yes	no	yes	n/a	yes 4000	n/a	n/a	yes	yes	n/a	n/a	yes	n/a	no	no	no
Logan F10.1	8	c.741 + 1G>A	homo	no	no	no	yes	n/a	yes 8900	n/a	n/a	no	yes	n/a	n/a	no	n/a	no	n/a	no
Logan F10.2	6	c.741 + 1G>A	homo	no	yes	yes	yes	n/a	yes 5000	n/a	n/a	no	yes	n/a	n/a	no	n/a	no	n/a	no
Wilton	3	c.161 + 1G>A; c.386G > C	compound hetero	no	yes	no	yes	no	yes 2755	yes	n/a	n/a	yes	n/a	n/a	yes	yes	no	yes	no
Lewis-Smith F1.1	5	5′UTR-Ex1del	homo	no	no	no	no	no	yes 2067	no	no	no	yes	no	no	no	no	no	no	yes
Lewis-Smith F1.2	6m	5′UTR-Ex1del	homo	no	yes	no	yes	no	yes 2000	no	yes	yes	no	no	no	no	no	no	no	yes
Roos	3	p.Q185X	homo	no	yes	yes	yes	n/a	yes	yes	n/a	n/a	no	n/a	n/a	yes	n/a	no	n/a	no
This case	1 y. 2m.	Ex2del	homo	no	no	no	no	yes	yes	yes	yes	yes	no	no	yes	no	no	no	no	yes

N/a, not available information in the text. F1, F2 etc – different families. F5.1, F5.2 etc – different patients in one family.

Here we present a patient with an unusual presentation of MICU1-associated myopathy without extrapyramidal signs due to homozygous deletion of exon 2.

## Materials and methods

### Subjects

The proband, a 3.5-year-old boy, and his parents underwent neurological examination and genetic investigation at the Research Centre for Medical Genetics, Moscow. Written informed consent was obtained from the family. The study was performed in accordance with the Declaration of Helsinki and approved by the Institutional Review Board of the Research Centre for Medical Genetics., Russia.

### Genetic analysis

Long deletions and duplications in *SGCA, SGCB, SGCG, SGCD*, and *FKRP* genes were analyzed using the commercially available kit SALSA MLPA P116 SGC probemix (MRC-Holland, The Netherlands) according to the original protocols. Data were analyzed with the original Coffalyser V8 software.

Deletion/duplication search in all 79 exons of the DMD gene was performed by the MLPA method using commercially available kits (MLPA SALSA P034 and P035 DMD probemix, MRC-Holland, The Netherlands), according to the manufacturer's recommendations. The reaction products were detected by fragmental analysis using an ABI Prism 3130 instrument (Applied Biosystems). Data analysis was carried out using Coffalyser.Net™ software.

Whole exome sequencing of proband DNA sample from peripheral blood was performed in-house (Genomed Ltd.) on Illumina NextSeq 500 instrument in 2 × 151 bp two paired-end modes to an average depth of 108.4×. The libraries were prepared and enriched using Illumina Nextera Rapid Capture Exome Kit v1.2; after read alignment, the corresponding target region list was used for sequencing depth calculation. The raw sequencing data had been processed with a custom pipeline based on popular open-source bioinformatics tools BWA, Samtools, and Vcftools, as well as in-house Perl scripts, using hg19 assembly as a reference sequence. Variant annotations were added by SnpEff/SnpSift software using public databases (dbSNP, ExAC, ClinVar, dbNSFP). Variants with a frequency greater than 0.01% were filtered out. All known pathogenic and LoF variants in the genes were analyzed first, followed by an analysis of splicing, missense, and synonymous variants. CNV analysis was performed using DELLY and Manta CNVnators. The cDNA and protein positions in MICU1 corresponded to transcript NM_001195518.2.

### RNA analysis of MICU1

Validation of the deletion and segregation analysis was performed using RT-PCR analysis. Total RNA was isolated from mononuclear cells with the standard Trizol-based method. cDNA was prepared with the ImPromII RT System (Promega) according to the manufacturer's recommendations. PCR was performed using primers specific for exons 1 and 4 of the MICU1 gene: MICU1_f1 (5'-GCTGCTGGAGCTCGTGTTT-3') and MICU1_r1 (5'-CCAGGCTCACTGATGACTTT-3'). PCR fragments were sequenced by Sanger sequencing on an ABI3130xl sequencer (Life Technologies) using the BigDye Terminator v1.1 Cycle Sequencing Kit.

## Results

### Clinical findings

The patient was followed up from the age of 2.5–3.5 in our center and was referred for the first time with elevated plasma CK level and three acute episodes of muscular weakness. He was born at 40 weeks of gestation from a second pregnancy in a healthy non-consanguineous family with no family history of inherited diseases. Delivery was normal: APGAR 8/9. Birth weight: 3360 g, length: 52 cm. The proband successfully reached early motor milestones but started to walk unsupported at 16 months.

The first episode of muscular weakness appeared at 14 months when he woke up. The family described the episode as mild muscular hypotonia. It was hard to get into a sitting position by himself and sit independently without support. The condition normalized in an hour.

The second episode occurred at 2 years and 2 months and was described as severe diffuse muscle weakness with no ability to sit or stand by himself followed by vomiting. The patient was admitted to the neurological department for further examination. An elevated CK level 4732–4068 U/L (<300 U/L), elevated ALT 86–102,7 U/L (<50 U/L), AST 109–181,9 U/L (<50 U/L), and LDH 515–586 U/L (72–182 U/L) were indicated during the examination in 2 weeks in the hospital. There were no specific changes on brain MRI or EEG or NCS.

The third episode occurred one month later and was described as severe diffuse muscle weakness. He lost the ability to hold his head, to sit without support, to stand up unsupported, and kept his eyes open. The boy was referred to the neurology department with suspected myasthenia. The neostigmine test had no effect. CK level was 2900 U/L. The surface EMG detected the mean amplitude of MUPs in lower limbs at 300 (μV), with velocity in n. tibialis 43 m/s with no decrement. Needle EMG was not performed. The motor skills were completely restored in 1.5 days.

During the clinical examination at 2 years 8 months, the boy had normal height, head, and chest circumferences. He was shy, but he tried to fulfill the proposed requests and had no learning difficulties or speech delay. There was slight weakness of the facial muscles, dyslalia, and elements of dysarthria. The gait was clumsy, with elements of waddling hips. He could jump but didn't fully lift his feet off the floor, the calf muscles were a little increased in volume and thickened. There was a lumbar hyperlordosis and signs of winged scapula were detected. While getting up he used support on his knee. Tendon reflexes were reduced, and symmetrical. There were no signs of ataxia or extrapyramidal. He had no special brain MRI features. He underwent the last neurological examination at 3.5 years and there were no additional clinical symptoms found.

### Molecular findings

First, Duchenne/Becker muscular dystrophy was suspected due to late unsupported walking, muscle weakness, elevated CK level, and light calf muscle hypertrophy. So, a targeted analysis of the *DMD* gene was performed. No deletions or duplications were found. Also, there were no CNVs detected in the targeted analysis of *SGCA, SGCB, SGCG, SGCD*, and *FKRP* genes for other frequent limb-girdle muscular dystrophies. Then, we performed a WES, and no plausible known pathogenic or other LoF SNVs in genes responsible for neuromuscular disorders were identified. But the analysis of exon coverage suspected possible homozygous deletion of exon 2 with unknown borders g. (?_74326370)-(74326571_?) in the *MICU1* gene.

**Figure 1:**
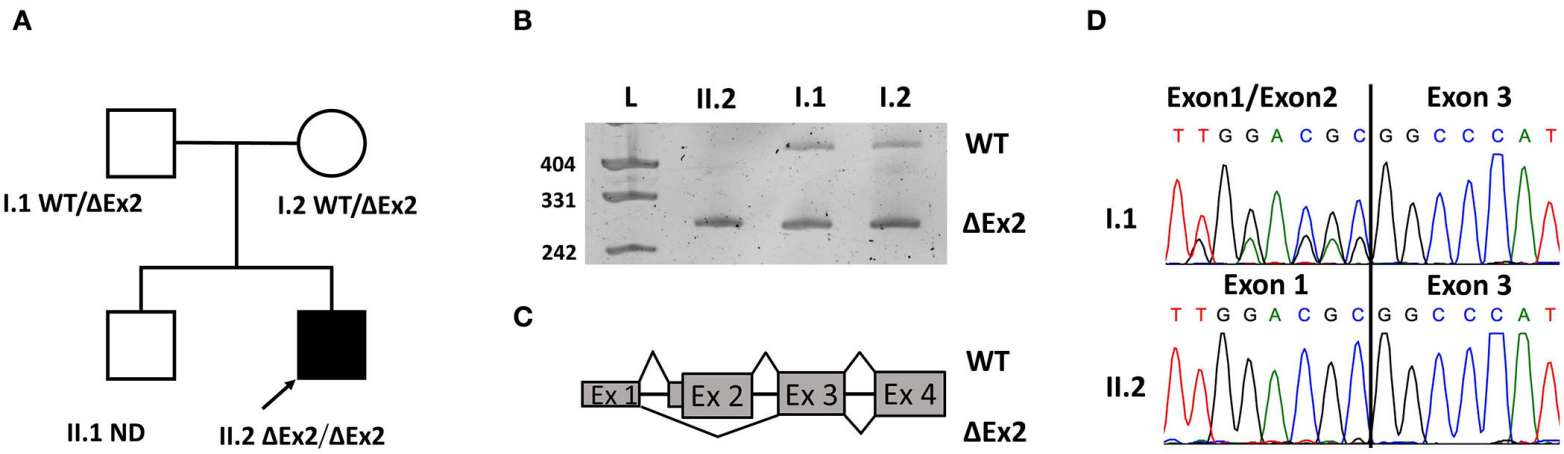
Pedigree of the family with genotypes **(A)**. Electropherograms of resulted products **(B)**. Scheme of exonic structure of WT transcript and transcript with the deletion of exon 2 (ΔEx2) **(C)**. Confirmation of WT and transcript ΔEx2 on Sanger sequences **(D)**.

The *MICU1* exon 2 is flanked by two large introns (about 60 and 4 kbp respectively with lots of repeats. So, it was impossible to validate the deletion with standard Sanger sequencing. Whole genome sequencing (WGS) or long-range PCR could be validation methods. But we decided to make it with an RNA analysis due to high *MICU1* gene expression in blood cells. To validate the deletion in the proband's sample and investigate its segregation through the family we amplified the locus containing exon 1 to exon 4 on the cDNA level with further Sanger sequencing. The ΔEx2 deletion in the homozygous state was confirmed in the patient's sample and found in the heterozygous state in his mother's and father's samples (Figure 1). Exon 2 is the first coding exon in the *MICU1* and the variant c.1_161del has to lead to a complete absence of translation (p.Met1?) from the start codon that it has to be pathogenic according to ACMG/AMP guidelines (PVS1, PM2).

## Discussion

Myopathies are a very heterogeneous group of diseases that often require multigene testing, such as WES. The efficiency of WES in childhood neurological conditions is about 20–53% according to the specificity of populations (11, 12).

In our case, we performed WES after a few targeted analyses for a patient with acute episodes of severe muscular weakness, elevated CK level, and mild myopathic features between the episodes. The homozygous ΔEx2 in the *MICU1* gene was suspected. The validation of such variants suspected by WES is difficult predominantly because of its size. The confirmation could be complicated by large introns or multiple intronic repeats. So, frequently WGS is the only way to find the exact borders of the CNV. Identification of exact coordinates is extremely important for preimplantation genetic diagnosis. But if we only need to confirm the presence of the deletion, then we can use an RNA analysis, which we did.

We successfully validated the deletion on a cDNA level in the homozygous state in the patient's sample and the heterozygous state in the mother's and father's samples. ΔEx2 led to a loss of the start codon in the *MICU1* gene and the protein does not have to translate from the affected allele, confirming the fact that the deletion is an LoF variant.

Also, it was interesting to detect a homozygous deletion in the non-consanguineous family according to the information from the family. We additionally investigated the places of birth of the proband's parents and did not find a similarity. It was hard to investigate older generations because of the lack of information in the family due to the huge impact of migration through the country.

LoF variants in the homozygous state led to the phenotype of *MICU1* progressive myopathy with extrapyramidal signs. The most common symptoms were motor delay (29/33), learning disabilities (28/31), elevated CK level (32/33), extrapyramidal signs (23/34), speech delay (19/31), muscle weakness (14/31), and elevated liver transaminases (14/17). Nine out of 30 had short stature, 7 out of 15 patients described muscular hypotonia, 7 out of 16 described an abnormal gait, 6 out of 20 had ataxia, and 5 out of 20 had microcephaly.

**Figure 2:**
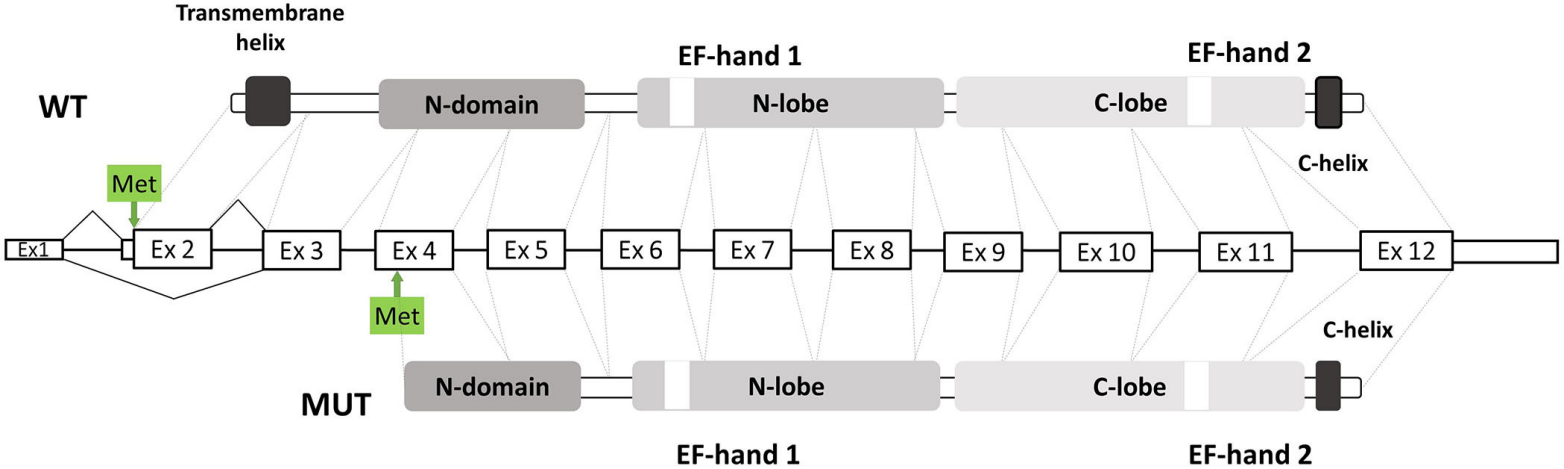
Scheme of exonic structure of WT MICU1 gene presents in the middle. Domain structure is shown in greys. Original ATG codon and predicted ATG codon shown with arrows and green boxes.

However, the natural history of the disease of our patient was different from previously reported ones (Table 1). To explain the unusual clinical picture, we suggested investigating the ΔEx2 impact on molecular pathogenesis. Using the in-silico ≪ATGpr≫ prediction algorithm we found a new Kozak sequence on the cDNA with the ΔEx2 (13). A new downstream ATG codon (chr10:74311096) showed a higher score, compared to the wild type one, and was placed in exon 4. Also, the translation from this alternative ATG codon is inframe with the major ORF of the *MICU1* gene. We supposed that ΔEx2 transcript could produce a new truncated protein p.Glu2_Met112del (Figure 2), but it was not functionally validated. Also, it was shown that MICU1 protein with a deletion of amino acids 61 to 130 could partially interact with the MCU channel, and the remaining EF1 and EF2 domains were necessary for the regulation of MCU-mediated Ca^2+^ uptake (14). So, p.Glu2_Met112del could be partly functional. The mitochondrial dysfunction was previously described for Duchenne muscular dystrophy (15) or RYR1-associated myopathies (16). Also, the highly variable clinical picture is distinctive for ion channelopathy (17, 18).

There is only one similar description of two cousins with acute episodes of the disease due to homozygous deletion of the 5'UTR region and non-coding exon 1 in the *MICU1*. Unfortunately, the authors described an absence of the MICU1 protein according to immunoblot assay, so the mechanism of the unusual clinical picture, in that case, could be different. A girl from the family had acute attacks of muscular fatigue and lethargy after minimum exercise from 5 years old. At 13 years she developed muscle aches after minimal walks. Her cousin described similar acute episodes in early childhood. At 12 years he developed migraine and vomiting episodes. Our patient also had acute episodes of muscular weakness with vomiting and mild myopathic features between the episodes, but he had no migraine or muscular aches after walks yet (10). Also, it is possible that the proband will develop these symptoms in the future.

## Conclusion

In conclusion, we demonstrate the ease and simplicity of using RNA analysis to confirm CNV, suspected by WES. Also, we present a case with the unusual presentation of *MICU1*-related myopathy due to homozygous ΔEx2 of the *MICU1* gene. We suggest that the main cause underlying the phenotype is the deletion of exons 2 leading to truncated MICU1 protein that still could interact with the MCU channel and regulates Ca^2+^ uptake into mitochondria.

We believe that this case could expand the spectrum of clinical phenotypes for *MICU1*-related myopathy.

## Data availability statement

The datasets presented in this article are not readily available because of ethical and privacy restrictions. Requests to access the datasets should be directed to the corresponding author.

## Ethics statement

The studies involving human participants were reviewed and approved by the Institutional Review Board of the Research Centre for Medical Genetics, Russia. Written informed consent to participate in this study was provided by the participants' legal guardian/next of kin. Written informed consent was obtained from the minor(s)' legal guardian/next of kin for the publication of any potentially identifiable images or data included in this article.

## Author contributions

All authors listed have made a substantial, direct, and intellectual contribution to the work and approved it for publication.

## Funding

The research was carried out within the state assignment of the Ministry of Science and Higher Education of the Russian Federation for RCMG.

## Conflict of interest

The authors declare that the research was conducted in the absence of any commercial or financial relationships that could be construed as a potential conflict of interest.

## Publisher's note

All claims expressed in this article are solely those of the authors and do not necessarily represent those of their affiliated organizations, or those of the publisher, the editors and the reviewers. Any product that may be evaluated in this article, or claim that may be made by its manufacturer, is not guaranteed or endorsed by the publisher.
